# Association Between Advanced Airway Management With Adrenaline Injection and Prognosis in Adult Patients With Asystole Asphyxia Out-of-hospital Cardiac Arrest

**DOI:** 10.2188/jea.JE20220240

**Published:** 2024-01-05

**Authors:** Kenichi Katabami, Takashi Kimura, Takumi Hirata, Akiko Tamakoshi

**Affiliations:** 1 Department of Public Health, Hokkaido University Graduate School of Medicine, Sapporo, Japan; 2 Department of Public Health, Hokkaido University Faculty of Medicine, Sapporo, Japan; 3 Institute for Clinical and Translational Science, Nara Medical University Hospital, Nara, Japan

**Keywords:** advanced airway management, asphyxia, out-of-hospital cardiac arrest

## Abstract

**Background:**

The neurological prognosis of asphyxia is poor and the effect of advanced airway management (AAM) in the prehospital setting remains unclear. This study aimed to evaluate the association between AAM with adrenaline injection and prognosis in adult patients with asystole asphyxia out-of-hospital cardiac arrest (OHCA).

**Methods:**

This study assessed all-Japan Utstein cohort registry data between January 1, 2013 and December 31, 2019. We used propensity score matching analyses before logistic regression analysis to evaluate the effect of AAM on favorable neurological outcome.

**Results:**

There were 879,057 OHCA cases, including 70,299 cases of asphyxia OHCAs. We extracted the data of 13,642 cases provided with adrenaline injection by emergency medical service. We divided 7,945 asphyxia OHCA cases in asystole into 5,592 and 2,353 with and without AAM, respectively. After 1:1 propensity score matching, 2,338 asphyxia OHCA cases with AAM were matched with 2,338 cases without AAM. Favorable neurological outcome was not significantly different between the AAM and no AAM groups (adjusted odds ratio [OR] 1.1; 95% confidence interval [CI], 0.5–2.5). However, the return of spontaneous circulation (ROSC) (adjusted OR 1.7; 95% CI, 1.5–1.9) and 1-month survival (adjusted OR 1.5; 95% CI, 1.1–1.9) were improved in the AAM group.

**Conclusion:**

AAM with adrenaline injection for patients with asphyxia OHCA in asystole was associated with improved ROSC and 1-month survival rate but showed no differences in neurologically favorable outcome. Further prospective studies may comprehensively evaluate the effect of AAM for patients with asphyxia.

## INTRODUCTION

The proportion of older adults is increasing worldwide, and the number of deaths due to asphyxia as a result of foreign body airway obstruction (FBAO) is also increasing in many countries.^1^^–^^4^ Approximately 120,000 cases of out-of-hospital cardiac arrest (OHCA) have been reported annually in Japan, and the survival rates are <10% in cardiac cases and <5% in non-cardiac cases.^5^ With respect to asphyxia, the neurological prognosis is known to be poor, and the favorable neurological outcome is <3% in asphyxia OHCA cases worldwide.^6^^–^^8^

In patients with asphyxia, the time to remove foreign bodies from the airway affects the survival rates and neurological prognoses.^7^^,^^9^^,^^10^ Igarashi et al reported in their recent multicenter retrospective study in Japan that prolonged airway obstruction was associated with poor neurological outcomes and that the goal should be to remove the airway foreign body within 4 minutes.^11^ In addition, previous reports in the United States have demonstrated that advanced airway management (AAM) and adrenaline injection by emergency medical service (EMS) were important to improve the OHCA outcomes.^12^^,^^13^ AAM, which comprises supraglottic airway (SGA) devices and endotracheal intubation (EDI), has been provided to patients with OHCA by EMS in Japan; however, its effect on the prognosis of patients with OHCA remains unclear.^14^^–^^23^ In some previous reports on AAM for asphyxia OHCA analyzed using the Utstein registry data, AAM was associated with increased risks for poor neurological outcomes.^24^^–^^26^ Otomune et al reported in their subgroup analyses that AAM with adrenaline injection for patients with asphyxia due to FBAO has no effect on neurological outcomes but can improve survival.^24^ AAM and adrenaline injection can be performed by EMS only for patients with cardiac arrest under the instruction of a medical doctor according to a local protocol in Japan.^27^ Especially, the patient in whom the adrenaline injection was administered to was definitely determined to be in cardiac arrest at that time.

Asphyxia is a condition caused by interference with respiration, due to which the organs and tissues are deprived of oxygen, causing unconsciousness or death. When asphyxia occurs, tachycardia and elevated blood pressure are first observed under the influence of stimulation of the respiratory center. Then, hypotension and bradycardia may occur, eventually leading to pulseless electrical activity to asystole.^28^ Cardiac arrest due to asphyxia implies a long clinical time course, meaning that there was prolonged hypoxic exposure to the brain and organs prior to cardiac arrest.^29^ This clinical course explains why the prognosis is worse in asphyxia than in shockable cardiac arrests.^30^ In general, asystole represents the terminal rhythm of a cardiac arrest. Asystole has been reported to be a sign that a long time has passed since the cardiac arrest occurred and that the patient has a poor prognosis.^31^ Experimental reports in dogs and rabbits have reported a gradual progression of bradycardia after asphyxia, eventually leading to asystole approximately 1 hour later.^32^^,^^33^ An experimental report in rats showed that the QRS waveform on electrocardiogram disappeared within approximately 10 minutes after asphyxia, and it took approximately 30 minutes to reach asystole.^28^

In a clinical emergency setting, we often experience situations in which we must choose AAM for patients with asphyxia OHCA in asystole. As emergency physicians, it is important to be familiar with the effects of AAM for patients with asphyxia OHCA in asystole and to inform the family in advance of the outcome. However, there have been few reports on the effect of AAM for such patients.

In this study, we aimed to evaluate the effect of AAM with adrenaline injection for patients with asphyxia OHCA in asystole on neurological outcomes.

## METHODS

### Study design and setting

We conducted a retrospective observational cohort study using all-Japan Utstein registry data of OHCA cases recorded by the Fire and Disaster Management Agency (FDMA) between January 1, 2013 and December 31, 2019. The details of this registry were previously reported.^34^ These data include almost all OHCAs that occurred in Japan, which are recorded in Utstein-style reporting templates by the FDMA.^35^ Moreover, these data are available to anyone with permission from the FDMA. Cardiac arrest was defined as the condition, in which the heart stopped mechanical beating and could no longer deliver effective circulation to the entire body and brain.^36^

We included patients with asphyxia OHCA who received adrenaline injection by EMS. We excluded those aged <18 and >100 years; those who did not receive adrenaline injection; those with time from call to cardiopulmonary resuscitation (CPR) or to hospital arrival of >60 minutes; and those who had missing data. We divided patients into two groups, namely the asystole and non-asystole groups, according to the initial ECGs because asystole suggested that a long period of time had passed in asphyxia OHCA. For patients with OHCA in Japan, EMS first performs chest compression and ventilation using a bag valve mask (BVM) and uses an automated external defibrillator (AED) as basic life support (BLS) according to the Japanese resuscitation guidelines.^27^ If patients cannot be adequately ventilated with BVM, the next option is to secure the airway with AAM, such as SGA devices and ETI; however, only specially trained EMS can perform ETI under medical control direction. Moreover, ETI is the treatment of choice in the following situations: (1) asphyxia due to FBAO, (2) absence of ventilation using an SGA device, and (3) requirement of an intubation per medical control doctor’s discretion. If necessary AAM and adrenaline injection are selected under medical control direction, which is permitted only for patients in cardiac arrest and not for those with ROSC. The requirement for informed consent was waived because all-Japan Utstein cohort registry data are public anonymized data and are publicly allowed to be analyzed. According to the Japanese ethical guidelines for medical and biological research involving human participants, the Ethics Review Committee of Hokkaido University Hospital determined that ethics review for this study was not required because the Utstein data were anonymous and had already been created.^37^

### Data collection and outcomes

The data prospectively collected from the all-Japan Utstein cohort registry of OHCA included age, sex, time of call for EMS, regional divisions, type of person witnessed, dispatcher’s instruction for CPR, BLS (chest compression, respiratory support, AED), initial ECG, AAM (SGA devices and ETI), adrenaline injection, time from call to CPR, time from call to hospital arrive, ROSC, 1-month survival, cerebral performance category (CPC) (1, good cerebral performance; 2, moderate cerebral disability; 3, severe cerebral disability; 4, coma or vegetative state; 5, death),^15^^,^^34^ and overall performance category (OPC) (1, normal; 2, mild disability; 3, moderate disability; 4, severe disability; 5, death). CPC was determined by the treating physician at 1 month after OHCA. In this study, the primary outcome was favorable neurological outcomes at 1 month after OHCA, which were defined as CPC 1 or 2. The secondary outcomes were ROSC and 1-month survival rates and OPC 1 or 2.

### Statistical analyses

We used propensity score (PS) matching analyses before logistic regression analysis to evaluate the effect of AAM on outcomes. We included the following variables, which were selected as important confounders in previous reports to estimate the PS to evaluate the effect of AAM: age, sex (male, female), dispatcher’s instruction for CPR (yes, no), bystander CPR of a healthcare provider (yes, no), time of call for EMS (0:00–5:59, 6:00–11:59, 12:00–17:59, 18:00–23:59), regional divisions (Hokkaido, Tohoku, Kanto, Chubu, Kinki, Chugoku and Shikoku, and Kyusyu and Okinawa), bystander BLS (push, respiratory, AED), and time from call to CPR. In the propensity matching analyses, 1:1 nearest matching between patients with and without AAM was conducted with a 0.02 caliper width.^6^^–^^11^ To evaluate propensity analyses, we used standardized mean differences (SMDs), which can be used to evaluate a balanced matching if it is <0.1.^38^ We calculated crude and multivariable adjusted odds ratios (ORs) and 95% confidence intervals (CIs) in logistic regression analyses to estimate the effect of AAM for primary and secondary outcomes. Continuous variables are presented as means and standard deviations (SDs) and as medians and interquartile ranges. Categorical variables are presented as numbers and percentages (%). We also analyzed patients with non-asystole OHCA using PS matching and logistic regressions to evaluate the effect of AAM for outcomes in non-asystole.

We used the EZR (version 1.55; Saitama Medical Center, Jichi Medical University) for all statistical analyses.

## RESULTS

**Figure 1.**
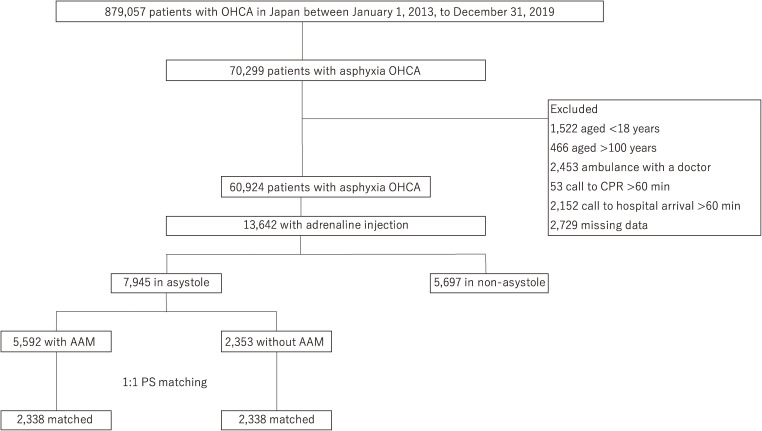
Flowchart of the study data documented in all-Japan Utstein out-of-hospital cardiac arrest registry data between January 1, 2013, and December 31, 2019. AAM, advanced airway management; CPR, cardiopulmonary resuscitation; OHCA, out-of-hospital cardiac arrest; PS, propensity score.

There were 879,057 OHCA cases, including 70,299 asphyxia OHCA cases documented in the all-Japan OHCA Utstein registry between January 1, 2013 and December 31, 2019 (Figure 1). After exclusion, 60,924 patients with asphyxia OHCA remained, and we extracted the data of 13,642 patients with adrenaline injection by EMS. We divided these patients into 7,945 with asystole and 5,697 with non-asystole. Finally, we divided patients with asphyxia OHCA with asystole into 5,592 and 2,353 patients with AAM and without AAM, respectively. After 1:1 PS matching, 2,338 patients with asphyxia OHCA with AAM and 2,338 such patients without AAM were included.

Table 1 shows the baseline characteristics of patients with asphyxia OHCA according to asystole on initial ECG. AAM was performed to 5,592 patients (70.4%) in asystole and 3,972 patients (69.7%) in non-asystole. Time from call to CPR was longer in the asystole than in the non-asystole group (9.8; SD, 3.5 min vs 9.3; SD, 3.5 min). Chest compressions were performed on 5,022 patients (63.2%) in asystole and 3,424 patients (60.1%) in non-asystole. Bystander CPR of a healthcare provider was performed to 110 patients (1.4%) in asystole and 370 patients (6.5%) in non-asystole. Favorable neurological outcomes were observed in 37 patients (0.5%) in asystole and 68 patients (1.2%) in non-asystole.

**Table 1.** Baseline characteristics of patients with asphyxia OHCA with adrenaline injection according to asystole

	Total	Asystole	Non-asystole
	*n* = 13,542	*n* = 7,945	*n* = 5,697
Age, years			
Median, (IQR)	82 (73–88)	82 (72–88)	83 (75–89)
Mean, (SD)	78.9 (14.0)	77.7 (15.2)	80.6 (11.9)
Sex			
Male, no (%)	7,463 (54.7)	4,355 (54.8)	3,108 (54.6)
Female, no (%)	6,179 (45.3)	3,590 (45.2)	2,589 (45.4)
Time of call for EMS			
0:00–5:59, no (%)	597 (4.4)	420 (5.3)	177 (3.1)
6:00–11:59, no (%)	3.936 (28.9)	2,264 (28.5)	1,672 (29.4)
12:00–17:59, no (%)	5.365 (39.3)	3,016 (38.0)	2,349 (41.2)
18:00–23:59, no (%)	3.744 (27.4)	2,245 (28.3)	1,499 (26.3)
Regional divisions			
Hokkaido, no (%)	915 (6.7)	644 (8.11)	272 (4.8)
Tohoku, no (%)	1.115 (8.2)	602 (7.6)	513 (9.0)
Kanto, no (%)	4.201 (30.8)	2.464 (31.0)	1.737 (30.5)
Chubu, no (%)	3.000 (22.0)	1.705 (21.5)	1.295 (22.7)
Kinki, no (%)	2.222 (16.3)	1.189 (15.0)	1.033 (18.1)
Chugoku, Shikoku, no (%)	1.063 (7.8)	672 (8.5)	391 (6.9)
Kyushu, Okinawa, no (%)	1.126 (8.3)	669 (8.4)	457 (8.0)
Bystander CPR of healthcare provider, no (%)	480 (3.5)	110 (1.4)	370 (6.5)
Dispatcher’s instruction for CPR, no (%)	9.298 (68.2)	5.489 (69.1)	3.809 (66.9)
Bystander BLS			
Chest compression, no (%)	8,446 (61.9)	5,022 (63.2)	3,424 (60.1)
Respiratory support, no (%)	1,213 (8.9)	668 (8.4)	545 (9.6)
AED, no (%)	123 (0.9)	74 (0.9)	49 (0.9)
Advanced airway management, no (%)	9,563 (70.1)	5,592 (70.4)	3.972 (69.7)
Time from call to CPR, min			
Median, (IQR)	9 (7–11)	9 (7–11)	9 (7–11)
Mean, (SD)	9.6 (3.5)	9.8 (3.5)	9.3 (3.5)
Time from call to hospital arrival, min			
Median, (IQR)	36 (30–42)	36 (31–43)	35 (30–42)
Mean, (SD)	36.5 (9.2)	36.9 (9.4)	35.9 (9.0)
CPC1–2, no (%)	105 (0.8)	37 (0.5)	68 (1.2)
ROSC, no (%)	4.780 (35.0)	2,453 (30.9)	2,327 (40.9)
1-month survival rate, no (%)	1.267 (9.3)	509 (6.4)	758 (13.3)
OPC1–2, no (%)	109 (0.8)	40 (0.5)	69 (1.2)

AED, automated external defibrillator; BLS, basic life support; CPC, cerebral performance category; CPR, cardiopulmonary resuscitation; EMS, emergency medical service; IQR, interquartile range; OPC, overall performance category; ROSC, return of spontaneous circulation; SD, standard deviation.

Table 2 shows the baseline characteristics of asphyxia OHCA in asystole according to AAM before and after PS matching analysis. After PS matching, 2,338 patients were matched for each group, and the baseline characteristics were well balanced between the AAM and no AAM groups except regional divisions (SMD <0.1).

**Table 2.** Baseline characteristics of patients with asphyxia OHCA in asystole according to AAM before and after PS matching analysis

	Before PS matching				After PS matching			

	Total	with AAM	without AAM		Total	with AAM	without AAM	
	*n* = 7,945	*n* = 5,592	*n* = 2,353	SMD	*n* = 4.676	*n* = 2.338	*n* = 2.338	SMD
Age, years				0.164				0.088
Median, (IQR)	82 (72–88)	82 (73–88)	81 (69–87)		80 (69–87)	79 (68–87)	81 (70–87)	
Mean, (SD)	77.7 (15.2)	78.4 (14.5)	75.9 (16.5)		75.4 (16.6)	74.7 (17.0)	76.2 (16.2)	
Sex				0.028				0.015
Male, no (%)	4,355 (54.8)	3,042 (54.4)	1,313 (55.8)		2,585 (55.3)	1.284 (54.9)	1.301 (55.6)	
Female, no (%)	3,590 (45.2)	2,550 (45.6)	1,040 (44.2)		2.091 (44.7)	1.054 (45.1)	1.037 (44.4)	
Time of call for EMS				0.040				0.038
0:00–5:59, no (%)	420 (5.3)	286 (5.1)	134 (5.7)		272 (5.8)	140 (6.0)	132 (5.6)	
6:00–11:59, no (%)	2,264 (28.5)	1,615 (28.9)	649 (27.6)		1.322 (28.3)	678 (29.0)	644 (27.5)	
12:00–17:59, no (%)	3,016 (38.0)	2,104 (37.6)	912 (38.8)		1.785 (38.2)	878 (37.6)	907 (38.8)	
18:00–23:59, no (%)	2,245 (28.3)	1,587 (28.4)	658 (28.0)		1.297 (27.7)	642 (27.5)	655 (28.0)	
Regional divisions				0.364				0.347
Hokkaido, no (%)	644 (8.1)	547 (9.8)	97 (4.1)		310 (6.6)	213 (9.1)	97 (4.1)	
Tohoku, no (%)	602 (7.6)	346 (6.2)	256 (10.9)		372 (8.0)	116 (5.0)	256 (10.9)	
Kanto, no (%)	2.464 (31.0)	1.818 (32.5)	646 (27.5)		1.381 (29.5)	735 (31.4)	646 (27.6)	
Chubu, no (%)	1.705 (21.5)	1.191 (21.3)	514 (21.8)		1.027 (22.0)	513 (21.9)	514 (22.0)	
Kinki, no (%)	1.189 (15.0)	857 (15.3)	332 (14.1)		649 (13.9)	317 (13.6)	332 (14.2)	
Chugoku, Shikoku, no (%)	672 (8.5)	474 (8.5)	198 (8.4)		451 (9.6)	257 (11.0)	194 (8.3)	
Kyushu, Okinawa, no (%)	669 (8.4)	359 (6.4)	310 (13.2)		486 (10.4)	187 (8.0)	299 (12.8)	
Bystander CPR of healthcare provider, no (%)	110 (1.4)	69 (1.2)	41 (1.7)	0.042	88 (1.9)	47 (2.0)	41 (1.8)	0.019
Dispatcher’s instruction for CPR, no (%)	5,489 (69.1)	3.902 (69.8)	1,587 (67.4)	0.050	3,104 (66.4)	1.524 (65.2)	1.580 (67.6)	0.051
Bystander BLS								
Chest compression, no (%)	5,022 (63.2)	3,546 (63.4)	1,476 (62.7)	0.014	2.827 (60.5)	1.359 (58.1)	1.468 (62.8)	0.095
Respiratory support, no (%)	668 (8.4)	468 (8.4)	200 (8.5)	0.005	407 (8.7)	209 (8.9)	198 (8.5)	0.017
AED, no (%)	74 (0.9)	58 (1.0)	16 (0.7)	0.039	30 (0.6)	14 (0.6)	16 (0.7)	0.011
Time from call to CPR, min				0.002				0.018
Median, (IQR)	9 (7–11)	9 (7–11)	9 (7–11)		9 (7–11)	9 (7–11)	9 (7–11)	
Mean, (SD)	9.8 (3.5)	9.8 (3.5)	9.8 (3.4)		9.8 (3.5)	9.7 (3.5)	9.8 (3.4)	

AAM, advanced airway management; AED, automated external defibrillator; BLS, basic life support; CPR, cardiopulmonary resuscitation; EMS, emergency medical service; IQR, interquartile range; OHCA, out-of-hospital cardiac arrest; PS, propensity score; SD, standard deviation; SMD, standardized mean difference.

Table 3 shows the crude and multivariable adjusted ORs of AAM in asystole for the primary and secondary outcomes after PS matching analysis. Regarding the primary outcome, favorable neurological outcome (CPC 1–2) was not significantly different between the AAM and no AAM groups (adjusted OR 1.1; 95% CI, 0.5–2.5). ROSC (adjusted OR 1.7; 95% CI, 1.5–1.9) and the 1-month survival rates (adjusted OR 1.5; 95% CI, 1.1–1.9) were significantly higher in the AAM than in the no AAM group, but the favorable functional outcomes (OPC 1–2) were not significantly different between the two groups (adjusted OR 1.0; 95% CI, 0.4–2.1).

**Table 3.** Crude and adjusted odds ratios of AAM for primary and secondary outcomes among asphyxia OHCA cases in asystole

	AAM					

	Yes	No	Crude OR	95% CI	Adjusted OR	95% CI
CPC1–2	14 (0.6)	12 (0.5)	1.2	0.5–2.5	1.1	0.5–2.5
ROSC	775 (33.1)	546 (23.4)	1.6	1.4–1.9	1.7	1.5–1.9
1-month survival rate	161 (6.9)	114 (4.9)	1.4	1.1–1.9	1.5	1.1–1.9
OPC1–2	13 (0.6)	13 (0.6)	1.0	0.5–2.2	1.0	0.4–2.1

AAM, advanced airway management; BLS, basic life support; CI, confidence interval; CPC, cerebral performance category; CPR, cardiopulmonary resuscitation; EMS, emergency medical service; OPC, overall performance category; OR, odds ratio; ROSC, return of spontaneous circulation.

adjusted factors: age, sex, time of call for EMS, regional divisions, bystander CPR of health care provider, dispatcher’s instruction for CPR, bystander BLS, time from call to CPR.

Table 4 shows the crude and multivariable adjusted ORs of AAM in non-asystole for the primary and secondary outcomes after PS matching analysis. Regarding the primary outcome, the favorable neurological outcomes (CPC 1–2) was not significantly different between the AAM and no AAM groups (adjusted OR 1.5; 95% CI, 0.8–2.9). ROSC (adjusted OR 1.6; 95% CI, 1.4–1.9) and 1-month survival (adjusted OR 1.7; 95% CI, 1.4–2.1) were significantly higher in the AAM than in the no AAM group, but favorable functional outcome (OPC 1–2) was not significantly different between the two groups (adjusted OR 1.6; 95% CI, 0.8–3.0).

**Table 4.** Crude and adjusted odds ratios of AAM for primary and secondary outcomes among asphyxia OHCA cases in non-asystole

	AAM					

	Yes	No	Crude OR	95% CI	Adjusted OR	95% CI
CPC1–2	24 (1.4)	17 (1.0)	1.4	0.8–2.7	1.5	0.8–2.9
ROSC	739 (42.9)	551 (32.0)	1.6	1.4–1.8	1.6	1.4–1.9
1-month survival rate	254 (14.7)	160 (9.3)	1.7	1.4–2.1	1.7	1.4–2.1
OPC1–2	23 (1.3)	16 (0.9)	1.4	0.8–2.7	1.6	0.8–3.0

AAM, advanced airway management; BLS, basic life support; CI, confidence interval; CPC, cerebral performance category; CPR, cardiopulmonary resuscitation; EMS, emergency medical service; OPC, overall performance category; OR, odds ratio; ROSC, return of spontaneous circulation.

adjusted factors: age, sex, time of call for EMS, regional divisions, bystander CPR of health care provider, dispatcher’s instruction for CPR, bystander BLS, time from call to CPR.

## DISCUSSION

In this Japanese nationwide Utstein registry cohort study with PS matching, we revealed that AAM with adrenaline injection for patients with asphyxia OHCA was associated with better 1-month survival and ROSC in both asystole and non-asystole patients. We also found that AAM with adrenaline injection for patients with asphyxia OHCA was not associated with poor neurological and functional outcomes. Especially in elderly patients, it is important to understand the effect of AAM for asphyxia OHCA in asystole, explain the prognosis to the family, and confirm their advance care planning before introducing AAM.

In previous reports, AAM for patients with asphyxia was associated with poor neurological outcomes.^24^^,^^25^ Unlike in previous reports, we did not find that performing AAM for patients with asphyxia OHCA in asystole had an opposite effect on favorable neurologic prognosis. One possible reason for this difference could be the different inclusion criteria for the group of patients for whom AAM was not performed. Especially, patients who were in cardiopulmonary arrest at the time of call but got ROSC before arrival of EMS and those who might have had a favorable outcome, may have been included in the previous studies. Sakurai et al reported that confirmed cardiac output on EMS arrival should be considered as a confounding factor, as indicated in observational studies of AAM.^39^ In the Japanese Utstein registry, the reasons for performing or not performing AAM for patients with OHCA were not recorded. Japanese EMS can only perform AAM and adrenaline injection for patients in cardiac arrest.^27^ If EMS determined that the patient was in cardiac arrest and adrenaline injection was performed, the patient was definitely in cardiac arrest at that time. Thus, in this study, we included only patients who had cardiac arrest, as determined by EMS, and received adrenaline injection.

Whether prehospital AAM is better than BVM for patients with OHCA remains unclear.^15^^,^^16^^,^^40^ According to Jabre et al in their randomized controlled trial of resuscitation for OHCA, there was no difference in neurological prognosis between ETI and BVM.^17^ Such reports have indicated the possibility of bias in the difficulty and success rate of the intubation technique, but no report has examined this issue in detail.^41^ According to Benger et al in their randomized controlled trial, in patients with OHCA, the type of AAM, such as ETI or SGA devices, did not change the outcome of neurological prognosis.^18^

Resuscitation time bias is common in Utstein registry analysis, which means that the resuscitation time affects the frequency of resuscitation interventions. Longer resuscitation duration is associated with worse outcomes and the later intervention is biased toward harmful.^42^ In this Utstein registry data, the exact onset time of the FBAO was not recorded. We believed that if we focused on asystole patients, we could focus on patients with severe asphyxia to reduce the influence of time bias as much as possible. Okubo et al reported that the timing of AAM was not statistically associated with improved 1-month survival for shockable rhythms, but AAM within 15 minutes after EMS-initiated CPR was associated with improved 1-month survival rates for non-shockable rhythms.^43^ Fukuda et al reported that delay in AAM was associated with a decreased chance of 1-month neurologically favorable survival among patients with OHCA.^44^ Izawa et al reported that in the time-dependent PS sequential matching for OHCA in adults, AAM was not associated with survival among patients with shockable rhythm, but AAM was associated with better survival among patients with non-shockable rhythm.^14^

Our study has several strengths. First, using nationwide Utstein registry data, this study could be conducted with a large sample size. Second, there were no studies concerning the effects of AAM with adrenaline injection for asphyxia OHCA focused on asystole, which indicated that a long time had passed and the condition was severe.

However, this study had some limitations. First, as the Utstein data were recorded only for determined items, we could not control many confounders in our study, such as medical histories, living conditions, activities of daily living, quality of BLS or ALS, detailed time course of resuscitation, detailed information of foreign bodies that obstruct patient’s airway, and medical treatment after hospital arrival. Second, this study used the Utstein data from Japan, and the results may not be simply applicable to other countries because of differences in the emergency medical care and EMS systems in these countries.^40^ Third, our study did not examine cases, in which intubation was technically impossible or difficult because the Japanese Utstein data do not provide detailed information on the reasons for the decision to intubate or not intubate. An increasing number of intubation attempts during OHCA resuscitation was associated with a lower likelihood of favorable neurological outcomes.^45^ Fourth, there is no information in the Ustein registry data to confirm whether the foreign body was removed from the airway. This may have implications for the effect of AAM in this study. Fifth, this study is a retrospective analysis of the Utstein data. Thus, there is a potential risk of power to detect the difference in the neurological outcome in this study. Finally, as the exact time of AAM was not recorded in the Ustein registry data, we could not analyze the resuscitation time bias. Further detailed studies, such as prospective randomized studies, on the effect of AAM for asphyxia are required.

In conclusion, AAM for asphyxia OHCA in asystole was associated with improved ROSC and 1-month survival rates, but it made no difference in the neurologically favorable outcomes. This result was different from that of previous studies, in which AAM had negative effects on the neurological outcomes of patients with asphyxia. Further prospective studies are expected to comprehensively evaluate the effect of AAM for patients with asphyxia.

## References

[1] Kramarow E, Chen LH, Hedegaard H, Warner M. Deaths from unintentional injury among adults aged 65 and over: United States, 2000–2013. NCHS Data Brief. 2015;199. 25973998

[2] Pavitt MJ, Nevett J, Swanton LL, . London ambulance source data on choking incidence for the calendar year 2016: an observational study. BMJ Open Respir Res. 2017;4:e000215. 10.1136/bmjresp-2017-000215 29299326 PMC5728301

[3] Chung CH, Lai CH, Chien WC, Lin CH, Cheng CH. A population-based study of inpatients admitted due to suffocation in Taiwan during 2005–2007. Accid Anal Prev. 2013;50:523–529. 10.1016/j.aap.2012.05.033 22717228

[4] Landoni G, Morselli F, Silvetti S, Frontera A, Zangrillo A; Collaborators. Pizza in adults and grape in children are the most frequent causes of foreign body airway obstruction in Italy. A national media-based survey. Resuscitation. 2020;149:141–142. 10.1016/j.resuscitation.2020.02.016 32114069

[5] FaDM Agency. *Status of EMS and Fire Rescue; Year Report of 2019*. Fire and Disaster Management Agency; 2019.

[6] Kiyohara K, Sakai T, Nishiyama C, . Epidemiology of out-of-hospital cardiac arrest due to suffocation focusing on suffocation due to Japanese rice cake: a population-based observational study from the Utstein Osaka Project. J Epidemiol. 2018;28:67–74. 10.2188/jea.JE20160179 29093354 PMC5792229

[7] Olasveengen TM, Mancini ME, Perkins GD, . Adult basic life support: International Consensus on Cardiopulmonary Resuscitation and Emergency Cardiovascular Care Science with Treatment Recommendations. Resuscitation. 2020;156:A35–A79. 10.1016/j.resuscitation.2020.09.010 33098921 PMC7576327

[8] Inamasu J, Miyatake S, Tomioka H, . Cardiac arrest due to food asphyxiation in adults: resuscitation profiles and outcomes. Resuscitation. 2010;81:1082–1086. 10.1016/j.resuscitation.2010.04.032 20627519

[9] Igarashi Y, Yokobori S, Yoshino Y, Masuno T, Miyauchi M, Yokota H. Prehospital removal improves neurological outcomes in elderly patient with foreign body airway obstruction. Am J Emerg Med. 2017;35:1396–1399. 10.1016/j.ajem.2017.04.016 28427784

[10] Norii T, Igarashi Y, Sung-Ho K, . Protocol for a nationwide prospective, observational cohort study of foreign-body airway obstruction in Japan: the MOCHI registry. BMJ Open. 2020;10:e039689. 10.1136/bmjopen-2020-039689 32690753 PMC7375623

[11] Igarashi Y, Norii T, Sung-Ho K, . Airway obstruction time and outcomes in patients with foreign body airway obstruction: multicenter observational choking investigation. Acute Med Surg. 2022;9:e741. 10.1002/ams2.741 35309267 PMC8918414

[12] Link MS, Berkow LC, Kudenchuk PJ, . Part 7: adult advanced cardiovascular life support: 2015 American Heart Association guidelines update for cardiopulmonary resuscitation and emergency cardiovascular care. Circulation. 2015;132:S444–S464. 10.1161/CIR.0000000000000261 26472995

[13] Kleinman ME, Brennan EE, Goldberger ZD, . Part 5: adult basic life support and cardiopulmonary resuscitation quality: 2015 American Heart Association guidelines update for cardiopulmonary resuscitation and emergency cardiovascular care. Circulation. 2015;132:S414–S435. 10.1161/CIR.0000000000000259 26472993

[14] Izawa J, Komukai S, Gibo K, . Pre-hospital advanced airway management for adults with out-of-hospital cardiac arrest: nationwide cohort study. BMJ. 2019;364:l430. 10.1136/bmj.l430 30819685 PMC6393774

[15] Granfeldt A, Avis SR, Nicholson TC, . Advanced airway management during adult cardiac arrest: a systematic review. Resuscitation. 2019;139:133–143. 10.1016/j.resuscitation.2019.04.003 30981882

[16] Wang HE, Schmicker RH, Daya MR, . Effect of a strategy of initial laryngeal tube insertion vs endotracheal intubation on 72-hour survival in adults with out-of-hospital cardiac arrest: a randomized clinical trial. JAMA. 2018;320:769–778. 10.1001/jama.2018.7044 30167699 PMC6583103

[17] Jabre P, Penaloza A, Pinero D, . Effect of bag-mask ventilation vs endotracheal intubation during cardiopulmonary resuscitation on neurological outcome after out-of-hospital cardiorespiratory arrest: a randomized clinical trial. JAMA. 2018;319:779–787. 10.1001/jama.2018.0156 29486039 PMC5838565

[18] Benger JR, Kirby K, Black S, . Effect of a strategy of a supraglottic airway device vs tracheal intubation during out-of-hospital cardiac arrest on functional outcome: the AIRWAYS-2 randomized clinical trial. JAMA. 2018;320:779–791. 10.1001/jama.2018.11597 30167701 PMC6142999

[19] Jeong S, Ahn KO, Shin SD. The role of prehospital advanced airway management on outcomes for out-of-hospital cardiac arrest patients: a meta-analysis. Am J Emerg Med. 2016;34:2101–2106. 10.1016/j.ajem.2016.07.025 27503061

[20] Benoit JL, Gerecht RB, Steuerwald MT, McMullan JT. Endotracheal intubation versus supraglottic airway placement in out-of-hospital cardiac arrest: a meta-analysis. Resuscitation. 2015;93:20–26. 10.1016/j.resuscitation.2015.05.007 26006743

[21] Tanabe S, Ogawa T, Akahane M, . Comparison of neurological outcome between tracheal intubation and supraglottic airway device insertion of out-of-hospital cardiac arrest patients: a nationwide, population-based, observational study. J Emerg Med. 2013;44:389–397. 10.1016/j.jemermed.2012.02.026 22541878

[22] Hasegawa K, Hiraide A, Chang Y, Brown DF. Association of prehospital advanced airway management with neurologic outcome and survival in patients with out-of-hospital cardiac arrest. JAMA. 2013;309:257–266. 10.1001/jama.2012.187612 23321764

[23] Kajino K, Iwami T, Kitamura T, . Comparison of supraglottic airway versus endotracheal intubation for the pre-hospital treatment of out-of-hospital cardiac arrest. Crit Care. 2011;15:R236. 10.1186/cc10483 21985431 PMC3334787

[24] Otomune K, Hifumi T, Jinno K, . Neurological outcomes associated with prehospital advanced airway management in patients with out-of-hospital cardiac arrest due to foreign body airway obstruction. Resusc Plus. 2021;7:100140. 10.1016/j.resplu.2021.100140 34223396 PMC8244501

[25] Ohashi-Fukuda N, Fukuda T, Yahagi N. Effect of pre-hospital advanced airway management for out-of-hospital cardiac arrest caused by respiratory disease: a propensity score-matched study. Anaesth Intensive Care. 2017;45:375–383. 10.1177/0310057X1704500314 28486897

[26] Fukuda T, Fukuda-Ohashi N, Doi K, Matsubara T, Yahagi N. Effective pre-hospital care for out-of-hospital cardiac arrest caused by respiratory disease. Heart Lung Circ. 2015;24:241–249. 10.1016/j.hlc.2014.09.004 25445432

[27] Japan Resuscitation Council. *Japanese Guidelines for Emergency Care and Cardiopulmonary Resuscitation*. Tokyo, Japan: Igaku Shoin; 2021; 2020.

[28] Hendrickx HH, Rao GR, Safer P, . Asphyxia, cardiac arrest and resuscitation in rats. I. Short term recovery. Resuscitation. 1984;12:97–116. 10.1016/0300-9572(84)90062-5 6091205

[29] Varvarousis D, Varvarousi G, Iacovidou N, . The pathophysiologies of asphyxia vs dysrhythmic cardiac arrest: implications for resuscitation and post-event management. Am J Emerg Med. 2015;33:1297–1304. 10.1016/j.ajem.2015.06.066 26233618

[30] Blondel M, Kohlhauer M, Ziberstein L, . How can we study cardiopulmonary resuscitation and cardiac arrest in animals: a review. J Dairy Vet Anim Res. 2016;3:37–41.

[31] Fukuda T, Fukuda-Ohashi N, Doi K, Matsubara T, Yahagi N. Association of initial rhythm with neurologically favorable survival in non-shockable out-of-hospital cardiac arrest without a bystander witness or bystander cardiopulmonary resuscitation. Eur J Intern Med. 2016;30:61–67. 10.1016/j.ejim.2016.01.022 26944563

[32] Saito Y. Studies on the electrocardiogram change in asphyxia. J Tokyo Women’s Med Univ. 1960;30:199–213 (in Japanese).

[33] Komura S, Fujimura K. Heart rate and fatal course in rabbits asphyxiated by respiratory arrest. Tohoku J Exp Med. 1974;114:273–275. 10.1620/tjem.114.273 4456687

[34] Kitamura T, Iwami T, Kawamura T, . Nationwide public-access defibrillation in Japan. N Engl J Med. 2010;362:994–1004. 10.1056/NEJMoa0906644 20237345

[35] Jerry PN, Robert AB, Lars WA, . Cardiac arrest and cardiopulmonary resuscitation outcome reports: update of the utstein resuscitation registry template for in-hospital cardiac arrest. Resuscitation. 2019;144:166–177. 10.1016/j.resuscitation.2019.08.021 31536777

[36] Perkins GD, Jacobs IG, Nadkarni VM, . Cardiac arrest and cardiopulmonary resuscitation outcome reports; update of the Utstein resuscitation registry templates for out-of-hospital cardiac arrest: a statement for healthcare professionals from a task force of the International Liaison Committee on Resuscitation (American Heart Association, European Resuscitation Council, Australian and New Zealand Council on Resuscitation, Heart and Stroke Foundation of Canada, InterAmerican Heart Foundation, Resuscitation Council of Southern Africa, Resuscitation Council of Asia); and the American Heart Association Emergency Cardiovascular Care Committee and the Council on Cardiopulmonary, Critical Care, Perioperative and Resuscitation. Circulation. 2015;132:1286–1300. 10.1161/CIR.0000000000000144 25391522

[37] Ministry of Education, Culture, Sports, Science and Technology, Ministry of Health, Labour and Welfare, and Ministry of Economy, Trade and Industry. Ethical guidelines for medical and biological research involving human subjects. https://www.mhlw.go.jp/content/000757566.pdf (4 December 2022, date last accessed) (in Japanese).

[38] Zhang Z, Kim HJ, Lonjon G, Zhu Y. Balance diagnostics after propensity score matching. Ann Transl Med. 2019;7:16. 10.21037/atm.2018.12.10 30788363 PMC6351359

[39] Sakurai A, Kinoshita K, Maeda Y, . Confirmed cardiac output on emergency medical services arrival as confounding by indication: an observational study of prehospital airway management in patients with out-of-hospital cardiac arrest. Emerg Med J. 2019;36:410–415. 10.1136/emermed-2018-208107 31171627 PMC6662946

[40] Oh YS, Ahn KO, Shin SD, . Variability in the effects of prehospital advanced airway management on outcomes of patients with out-of-hospital cardiac arrest. Clin Exp Emerg Med. 2020;7:95–106. 10.15441/ceem.19.022 32635700 PMC7348675

[41] Lupton JR, Schmicker RH, Stephens S, . Outcomes with the use of bag-valve-mask ventilation during out-of-hospital cardiac arrest in the pragmatic airway resuscitation trial. Acad Emerg Med. 2020;27:366–374. 10.1111/acem.13927 32220129

[42] Andersen LW, Grossestreuer AV, Donnino MW. “Resuscitation time bias”—A unique challenge for observational cardiac arrest research. Resuscitation. 2018;125:79–82. 10.1016/j.resuscitation.2018.02.006 29425975 PMC6080954

[43] Okubo M, Komukai S, Izawa J, . Timing of prehospital advanced airway management for adult patients with out-of-hospital cardiac arrest: a nationwide cohort study in Japan. J Am Heart Assoc. 2021;10:e021679. 10.1161/JAHA.121.021679 34459235 PMC8649292

[44] Fukuda T, Ohashi-Fukuda N, Inokuchi R, . Association between time to advanced airway management and neurologically favourable survival during out-of-hospital cardiac arrest. Anaesth Crit Care Pain Med. 2021;40:100906. 10.1016/j.accpm.2021.100906 34147685

[45] Murphy DL, Bulger NE, Harrington BM, . Fewer tracheal intubation attempts are associated with improved neurologically intact survival following out-of-hospital cardiac arrest. Resuscitation. 2021;167:289–296. 10.1016/j.resuscitation.2021.07.001 34271128

